# Capturing Features and Performing Human Detection from Human Gaits Using RFID

**DOI:** 10.3390/s22218353

**Published:** 2022-10-31

**Authors:** Yajun Zhang, Xu Liu, Zhixiong Yang, Zijian Li, Xinyue Zhang, Bo Yuan

**Affiliations:** 1 School of Software, XinJiang University, Ürümqi 830091, China; 2 School of Information Engineering, Shanghai Maritime University, Shanghai 201306, China

**Keywords:** deep learning, person identification, RFID, target detection

## Abstract

Recently, radio frequency identification (RFID) sensing has attracted much attention due to its contact-free nature, low cost, light weight and other advantages. RFID-based person detection has also become a hot research topic, but there are still some problems in the existing research. First, most of the current studies cannot identify numerous people at a time well. Second, in order to detect more accurately, it is necessary to evaluate the whole-body activity of a person, which will consume a lot of time to process the data and cannot be applied in time. To solve these problems, in this paper we propose RF-Detection, a person detection system using RFID. First of all, RF-Detection takes step length as the standard for person detection, divides step length into specific sections according to the relationship between step length and height, and achieves high accuracy for new user detection through a large amount of training for a specific step length. Secondly, RF-Detection can better identify the number of people in the same space by segmenting continuous people. Finally, the data collection was reduced by expanding the data set, and the deep learning method was used to further improve the accuracy. The results show that the overall recognition accuracy of RF-Detection is 98.93%.

## 1. Introduction

In recent years, with the rise of passive sensing technology, person detection technology plays an important role in people’s life and work, whether in the epidemic of COVID-19 or in the rapid development of intelligent technology for the future, such as overcrowding detection, road crowding detection, person detection at disaster relief sites, etc. At the same time, researchers are also committed to realizing more fine-grained passive sensing activities, applying passive sensing to public places with large crowds, such as shopping malls, offices, parking lots, and stations, in addition to special scenes, which enables person detection technology to enter people’s daily life.

Traditional person detection often uses infrared, ultrasonic, or camera monitoring, and Wi-Fi and other technologies. Although these methods have high recognition accuracy, they have great limitations in the more common multipath environment. For example, infrared technology [1] is usually used in indoor spaces with few multipath paths. When people pass, they will trigger the corresponding alarm. However, infrared technology is often used for straight line transmission, and its accuracy will be affected when there are obstructions. Ultrasonic technology [2] has high sensitivity, but its equipment requirements are high, and it is rarely used in multipath environments. Camera surveillance is a common device in our life, which can monitor people’s motion in real time, but this technology cannot play a very good role in an environment with weak visibility. Wi-Fi technology [3] also plays a good role in person detection due to its cheap price and wide signal coverage. However, in complex multipath indoor person detection, it is not able to comprehensively cover corners, edges and other environments, so it is usually necessary to add more APs to achieve comprehensive coverage.

Recently, person detection systems based on radio frequency identification (RFID) have attracted a lot of research interest because of their passive and contact-free characteristics. This means that the user does not need to carry any device to carry out the person detection, and there is no risk of violating the user’s privacy. Although RFID has shown considerable advantages in person detection, these identifications still have multiple limitations to some extent, which hinders the popularization of RFID in daily life. First of all, most systems collect human gait data by using the same walking pattern for a human in a normal state, but this is not always the case in real life. Even for the same user, due to changes in his mood or state, his walking style is always changing, and the data for a single state cannot be used as the standard, which leads to a failure to detect complete and stable gait data. Secondly, it takes a lot of manpower to obtain the gait data of each person, and a large amount of data is needed for training to support the expected results, which is very time consuming. Thirdly, most existing systems focus on personnel recognition, which can accurately identify the characteristics of each person, but also requires the user to walk in a certain posture and cannot accurately identify when multiple people pass through continuously. Most systems cannot be applied well in life.

In order to solve the above problems, we propose the person detection system RF-Detection, which is based on RFID. Compared with traditional technologies, ultra-high-frequency (UHF) RFID tags are inexpensive, small, flexible, and can transmit energy without the need for additional equipment. As shown in Results, we place the tag group antenna face-to-face, and the user walks between the tag group and the antenna. RF-Detection will map the captured signal to the gait feature executed. There are three challenges in designing the RF-Detection system.

Challenge 1: RF-Detection obtains unique characteristics of user gait data from RFID tags, which will often change with changes in users’ emotions and walking patterns. Moreover, due to the multipath effect of the environment, additional noise interference will be caused to the collected data, making it difficult to carry out accurate and effective identification. Therefore, these characteristics need to be supported by a more robust system, and the data should be more stable. How can we detect users more accurately when they are walking? To tackle this challenge, we use multiple RFID tags to extract more robust user gait data by extending the walking distance. In order to prevent interference from upper body movements such as the hands, we repeatedly tested the tag position to make it highly uniform and reduce the influence on the received signal. Finally, in order to obtain stable gait features, we designed a neural network module for training.

Challenge 2: How can we use limited data to detect new users without retraining? To tackle this challenge, we first divided the step length of the user group. From [4], we can see that the step length has a strong correlation with height. After that, the step characteristics of different populations were collected by experiments, which reduced the amount of data collection to a certain extent. Then, we used the method of data augmentation to further expand the data set, so as to reduce the data collection work. Finally, we fine tune the neural network to adapt the trained network to new user recognition.

Challenge 3: How can we accurately detect the specific number of users when multiple users pass through in succession? In order to solve this problem, by comparing the data of a single user, we automatically segmented the continuous data collected by RFID electronic tag to adapt to the multi-user scene.

We use COTS UHF RFID devices (five tags and one reader) to implement our system and evaluate the system performance in environments with different multipath effects. Extensive experiments demonstrated that RF-Detection has an average accuracy of 98.93% for human detection. The main contributions of this paper are as follows:

1. As far as we know, this paper is the first attempt to use step length as the main feature of human gait for RFID person detection.
2. We propose an ingenious user group grouping solution based on the relationship between people’s height and step length. By detecting users with different step lengths, we can effectively reduce the amount of data and make data more robust and accurate. We also use deep learning methods as well as continuous user data segmentation to obtain rich features for multi-user detection.
3. The effectiveness and robustness of RF-Detection are proved by a large number of practical experiments.

## 2. Materials and Methods

### 2.1. Related Work

Related work is divided into the following research areas: person detection technology and RFID-based sensing.

#### 2.1.1. Person Detection Technology

Recently, person detection technology has become an important research direction and is used in various fields [5,6,7]. Traditional person detection methods are based on wearable sensors [8,9] or cameras [10,11,12]. The use of these techniques can demonstrate excellent detection capabilities, but these approaches still have privacy concerns and cost concerns. In recent years, RFID, as a very promising technology, has shown excellent performance in many fields [13,14], and it is completely feasible to use RFID for person detection. On the one hand, it has the characteristics of convenience and low cost; on the other hand, it has a good privacy protection effect, and can more effectively protect user information in person detection. In [15], the author proposed a person detection method using RFID which can be better applied to privacy scenes. Specifically, [16] proposed a non-contact human activity detection method which greatly increased the detection area by using a cross-circular polarization configuration between reader antennas and tag antennas. In contrast, RF-Detection combines person detection with human height–step length relationship, it takes step length as the main indicator in data collection, and it divides the data into five groups according to height (see the experiment section for details), which greatly reduces the complexity of data collection and classification. In addition, it can more accurately estimate the height range of new users and detect the number of users in a specific area according to their step length signals.

#### 2.1.2. RFID-Based Sensing

RFID has been widely used in various innovative fields. For example, [17,18] used RFID to identify target materials, especially for liquids. In [19], RFID was used for vehicle positioning without Global Positioning System (GPS), and the Chinese Remainder Theorem (CRT) was used to solve the problem reasonably. At the same time, RFID can also complete more complex tasks, such as activity recognition [20], human body recognition [21], vital signs monitoring [22,23], personnel positioning [24] and so on. Lili Chen et al. [25] performed respiratory monitoring with a commercial RFID device by detecting signal fluctuations at the receiver caused by chest displacement during breathing. They also used optimization technology to locate multiple RFID tags so that the system can monitor the respiration of two target persons. Xingyu Chen et al. [26] used RFID to achieve item level temperature sensing. By observing the current changes around the tag circuit, ordinary passive tags are used as thermometers. In addition, [27] realized multi-object tracking based on RFID with the aid of vision, combined computer vision and RFID technology with a pair of correctly deployed RFID antennas and cameras, and proposed a new recognition mode which can be used together with image-level trajectory information. Chao Feng et al. [28] used the vertical arrangement of multiple tags to identify different persons through RFID and make a detailed distinction between dynamic and static persons, while Anna Huang et al. [29] used tag array to capture the distinguishing features between individuals for device-free user identification. Xiaoyi Fan et al. [30] extracted the signal angle-of-arrival (AOA) information from the multi-antenna array to realize device-free activity identification. Lin Feng et al. [31] identified sitting posture by pasting labels on people’s backs.

This paper focuses on building a contact-free person detection system using RFID, which can capture the gait information of all people in the current range and estimate their height and the number of people. Different from the above works, this paper no longer uses complex tag array to obtain human features, but uses fewer tags to obtain human gait information in a specific combination way and takes step length as an important standard for people detection. In addition, the number of people in the area can only be obtained from gait features without visual assistance, which greatly reduces the cost. According to the experimental data, RF-Detection can well distinguish people with different step lengths and it has a good performance in the identification of continuous people.

### 2.2. Preliminaries

In this section, we first introduce the RFID technology used in this paper. Then a preliminary study is conducted to show the adaptability of volunteers to the experiment.

#### 2.2.1. RFID Principle

A typical RFID system consists of three parts: reader, antenna and tag. Moreover, in RFID technology, as a medium to perceive the target environment, the tag can be divided into three kinds: active, semi-active and passive. In a passive RFID system, tags can work without batteries. The reader sends electromagnetic waves of a certain frequency through the antenna. When the tag is within the working range of the transmitting antenna, it receives electromagnetic waves from the antenna to activate its own circuit and sends out backscattering signals. The antenna transmits the received backscattered signal from the tag to the reader, which demodulates and decodes the signal and then sends it to the server for further processing. The signal received by the reader, namely the backscattered signal $S\left(t\right)$, can be expressed as:

(1) $$S\left(t\right)=a\left(t\right)e^{-j\theta \left(t\right)}$$

(2) $$\theta \left(t\right)=\left(\frac{2d}{\lambda }\times 2\pi +\mu \right)\mathrm{mod}\;2\pi \;$$

where $a\left(t\right)$ and $\theta \left(t\right)$ are the amplitude and phase of the backscattered signal, j is an imaginary unit and d is the distance between the antenna and the tag. Since the tag will generate backscatter signal after receiving the signal from the antenna, the actual propagation distance should be 2d. μ is the initial offset caused by the device, including the phase shift caused by the reader, tag and antenna. λ is the wavelength of the RF signal.

**Figure 1:**
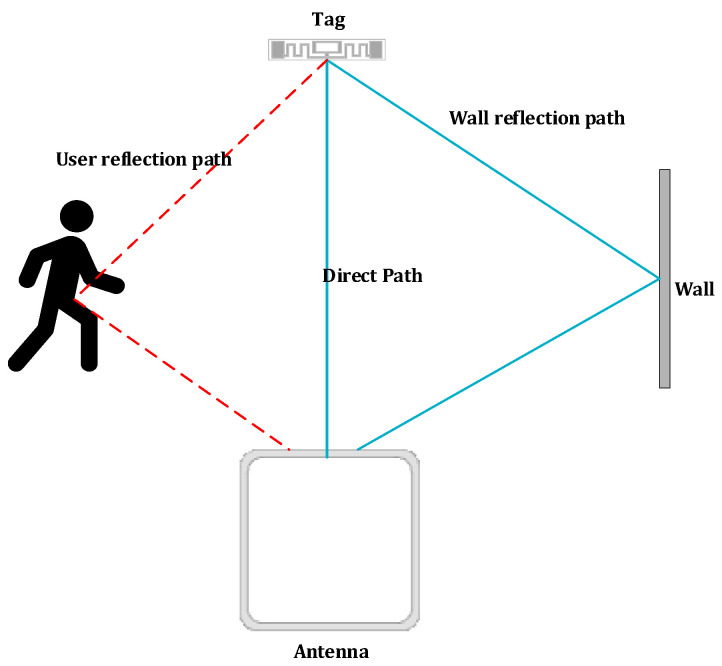
Signal propagation path.

When the user passes through the detection area, the signal will be propagated in three directions, as shown in Figure 1. This includes direct paths, obstacle reflection paths and dynamic reflection paths as the user moves. The backscattering signal $S\left(t\right)$ is the superposition of dynamic and static reflection signals, the static reflection signal is the composite signal composed of the direct path and the obstacle reflection path, and the dynamic reflection signal is the reflection signal when the user moves. When the user moves between the antenna and the tag, assuming that there is a total of n reflection paths of the user, the received signal $S\left(t\right)$ can be expressed as:

(3) $$S\left(t\right)=S_{s}\left(t\right)+S_{d}\left(t\right)\;\;$$

(4) $$S_{d}\left(t\right)=\underset{n}{\sum }a_{n}e^{-j\left(\frac{2\pi }{\lambda }\int d_{n}\left(t\right)dt+\mu \right)\mathrm{mod}\;2\pi }\;$$

(5) $$S_{s}\left(t\right)=a_{s}e^{-j\theta_{s}}\;$$

where $S_{s}\left(t\right)$ is the static reflected signal, $S_{d}\left(t\right)$ is the dynamic reflected signal when the user moves, $a_{n}$ is the amplitude of the user in the $n{\;}^{th}$ path, and $d_{n}\left(t\right)$ is the propagation distance at time t in path n.

**Figure 2:**
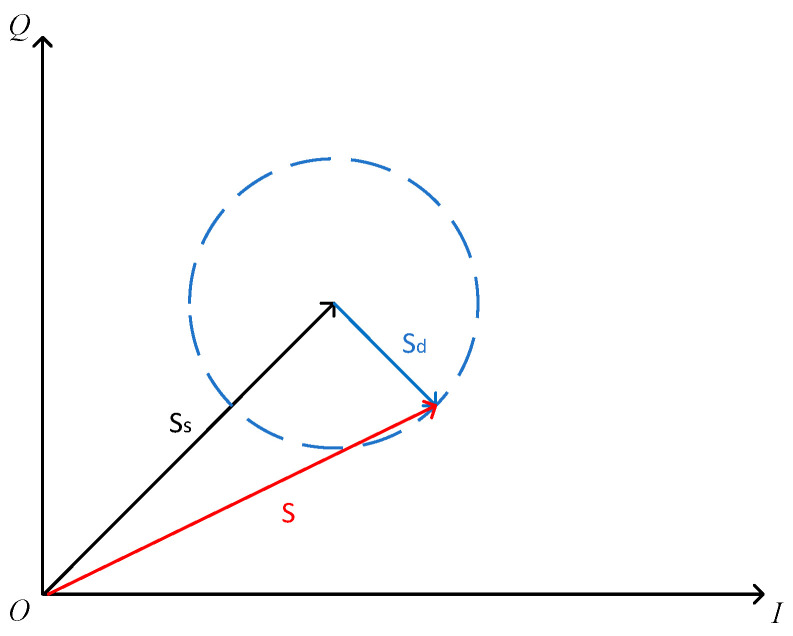
The signal decomposition model.

When the user moves the antenna-tag directly, the dynamic reflected signal will fluctuate accordingly. As shown in Figure 2, in the process of moving, $S_{d}$ will rotate relative to $S_{s}$, and the phase and amplitude of the corresponding synthesized signal S will also change accordingly. This means that the phase and amplitude information of the synthesized signal can be used for user detection. Specifically, on the one hand, we use phase and amplitude to obtain whether the user exists or not. On the other hand, we set multiple labels to enhance the user’s detection ability and reduce the detection blind area.

#### 2.2.2. Preliminary Experiment and Analysis

**Figure 3:**
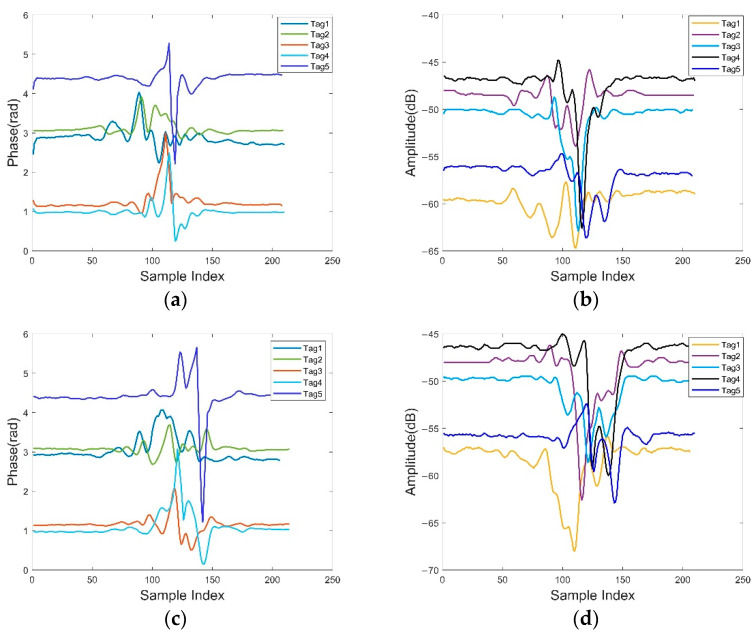
Phase and amplitude readings of two asynchronous volunteers under all five tags: (**a**,**b**) are the phase and amplitude of volunteer 1; (**c**,**d**) are the phase and amplitude of volunteer 2.

In order to better show the correlation between user step length and height and the influence of multiple users, we conducted two preliminary experiments. The five tags and antennas are deployed on both sides of the corridor. The labels are placed in a row parallel to the antenna. The width of the corridor is 2 m, the label spacing is 0.7 m and the label is about 0.3 m above the ground. The receiving range of the antenna is about 120°. In this experiment, height cannot be used as the evaluation standard for RF-Detection. Therefore, we combine the relationship between height and step length, and grouped step length to take it as the detection standard. Compared with single tag, multi-tag placement can largely cover all signal ranges for a long corridor, and more effectively capture the behavior characteristics of users with different heights and different step lengths. In the first experiment, we first analyzed the relationship between user height and step length. According to a large amount of data, different walking speed, stride frequency and walking type may have a certain impact on the relationship between height and step length, which is clearly shown in Challenge 2, where detailed calculation methods are given. In the experiment, since the walking speed of the experimenter was maintained in a relatively stable condition (i.e., normal walking), the linear relationship between the height and the step length in [32] was adopted for experimental analysis. We had two volunteers with different height–steps walk through a corridor sensing area. Figure 3 depicts the preprocessed phase and amplitude data of the two volunteers. The detailed processing process is shown in Section 2.3. We can see that different tags have different phase and amplitude changes, especially when volunteers pass through tag 3, where the change is most obvious. In addition, the volunteers who were out of sync also caused changes in phase and amplitude. These findings indicate that the phase and amplitude of tags imply physical features such as spatio-temporal correlation and behavioral features, which can be used for human detection.

**Figure 4:**
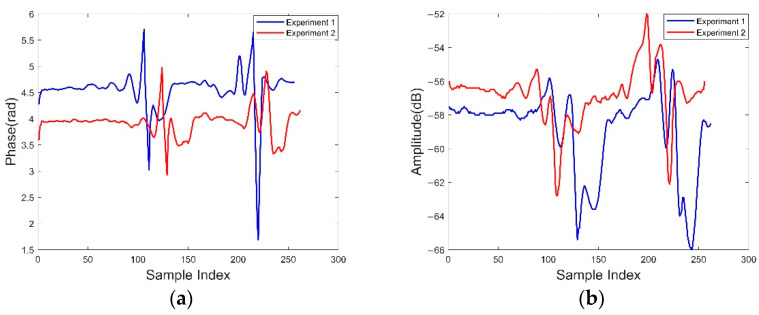
Phase and amplitude of two volunteers as they pass continuously: (**a**) Phase presentation of two volunteers under different experiments; (**b**) Amplitude presentation of two volunteers under different experiments.

In order to detect multiple users, in the second experiment we had two volunteers with different step lengths and two volunteers with the same step length continuously walk through the corridor detection area. Figure 4 shows two different sets of phase and amplitude readings. From the two groups of different phase values, it can be seen that the phase data of the two volunteers present a continuous state, and this state can be identified by segmentation, which proves that the RF-Detection system has good detection results in dealing with continuous person detection.

### 2.3. System Design

In this section, we first introduce the system design of RF-Detection in Section 2.3.1 and then detail the core modules of the system later.

#### 2.3.1. System Overview

**Figure 5:**
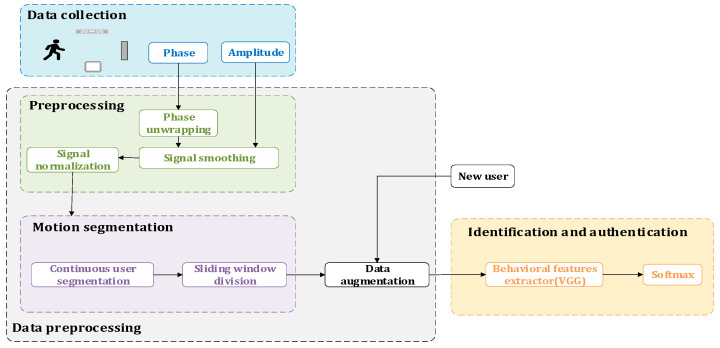
RF-Detection System.

As shown in Figure 5, RF-Detection is mainly composed of three modules: data collection module, signal preprocessing module and deep learning module.

**Figure 6:**
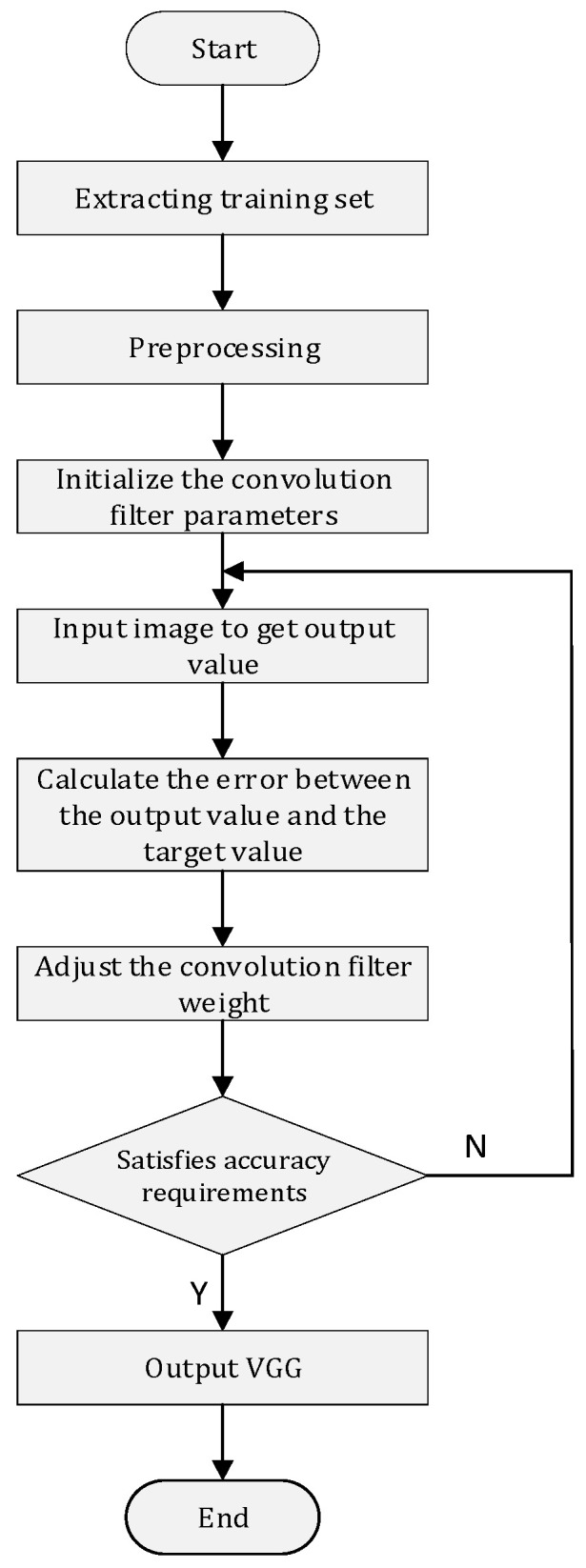
VGG training process.

First, we obtain the raw phase and Received Signal Strength Indicator (RSSI) data captured by the RFID tag through the antenna. After that, we perform preprocessing operations on the data. In addition to conventional phase unwrapping, normalization and other operations, we also perform segmentation and processing on the human motion signal, and we expand the data and other operations to improve the robustness and diversity of the data. After data preprocessing, it will be sent to the deep learning module. Specifically, this module is mainly composed of a Visual Geometry Group (VGG) neural network. The preprocessed data is sent into the module to extract the walking behavior of the persons first, so as to obtain their walking characteristics, and constantly adjust the weight of the convolution filter to achieve satisfactory results. Finally, the classification and identification are carried out through the output layer, with the specific process shown in Figure 6.

#### 2.3.2. Signal Preprocessing

Considering the interference of environmental noise and other factors, it is not reliable to operate directly on the raw signal; therefore, a series of preprocessing operations need to be performed to improve the signal recognition ability.

**Figure 7:**
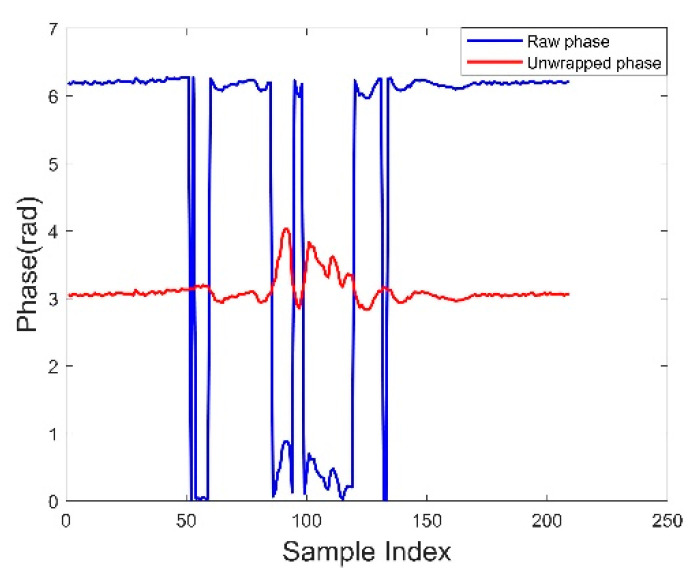
Comparison before and after phase unwrapping.

*Phase unwrapping.* The RFID signal phase reported by the RFID reader is a periodic function from 0 to 2π, also known as the wrapped phase. This wrapping phase will affect the subsequent analysis process due to its ambiguity. When the phase changes, it may jump from 2π to 0 or decrease to 0, so the absolute difference between two adjacent readings should be less than π to be reasonable. Therefore, it is necessary to unwrap the phase. We adopt one of the phase unwrapping methods in [33] and the results are shown in Figure 7.

**Figure 8:**
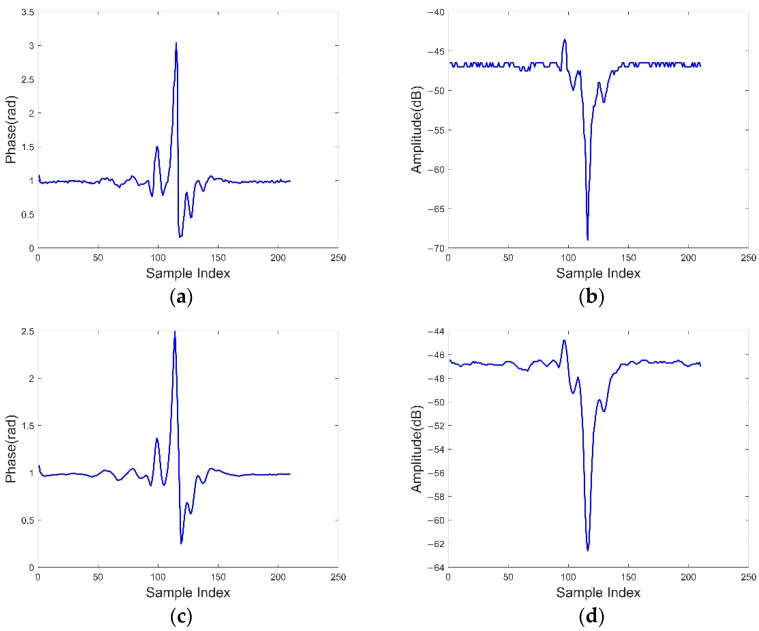
Phase and amplitude before and after data smoothing: (**a**,**b**) show the raw phase and amplitude; (**c**,**d**) show the phase and amplitude after processing.

*Signal smoothing.* Due to the environmental noise and the defects existing in the hardware itself, the collected raw phase and amplitude are often affected by noise, as shown in Figure 8a,c. Therefore, we use the Savitzky–Golay Filter (SG Filter) [34] for smoothing. The SG filter is usually used to smooth a set of data and is able to significantly improve the accuracy of the data without changing the trend and width of the signal. The main principle of the filter is to fit a continuous subset of adjacent data points to a low-degree polynomial by the linear least squares method, which shows obvious advantages when dealing with time series data. Therefore, the SG filter is more applicable in RFID with data changes. The phase and amplitude after denoising are shown in Figure 8b,d.

*Signal normalization.* In RFID readers, the collected raw phases may have different dimensional units, which will reduce the comparability between the data and reduce the accuracy of person detection. In order to ensure reliable and accurate results, we normalize the data to improve the convergence speed and accuracy of the model. Specifically, we use min-max normalization [35] to linearly transform the original data x and output y:

(6) $$y=\frac{x-min}{\max-min}$$

where min and max are the minimum and maximum values in the set, respectively. In addition, the reason why min-max normalization is selected instead of Z-score normalization is that the convergence speed of min-max normalization and the improvement effect on accuracy are better.

*Motion segmentation.* After normalization and smoothing, we obtain a relatively smooth curve. At this time, in order to obtain effective motion segments, this curve needs to be segmented and processed. Because the phase and amplitude fluctuations vary greatly between different step lengths, and the segments of continuous human motion are difficult to separate, it is difficult to obtain motion segments by threshing-based segmentation methods. At the same time, due to the influence of environment and subjective consciousness, the setting of ordinary threshold may lead to a significant decline in the performance of mixed activities. In order to deal with these problems, inspired by the method in [36], we first segment the motion curve of continuous persons through its data volatility, and then use the sliding window segmentation method based on variance.

*Continuous human motion segmentation.* First, we use an average slope k to represent the volatility of the entire data segment, and we calculate the standard deviation σ of the entire data. Then, we use the idea of moving average to eliminate the low-frequency noise. After the new data segment is obtained, the volatility of the data is judged; when the data fluctuation is greater than σ, the data is beat, the current corresponding index value is recorded, and then the search continues backward; when other values are also greater than σ, the index value is refreshed; if not, the judgment is over, and the current index value is recorded. For the current index value, it is necessary to further determine whether the index is generated by gait or environmental interference, so we set the action to be valid when the maximum fluctuation value in the intercepted signal is greater than 1.5 σ. Finally, the start and end indexes of the data were saved to complete the segmentation task.

*Sliding window.* For the partitioned gait data, we need to perform gait detection and segmentation for each region. From Figure 8, we learn that the signal has obvious fluctuations when there are gait features, while the signal tends to a stable state when there are no gait features. In addition, each set of complete gait data contains four states: stillness–start–movement–end. Therefore, we detect gestures by using variance. We set a time window T of length [Δt−T,Δt+T] within each region Δt, where T=0.2 s. Then we calculate the average standard deviation $\sigma_{\Delta t}$ at Δt, and choose the smallest $\sigma_{\min}$ as the variance threshold. The sliding window rule is as follows:

(7) $$\sigma_{\Delta t}=\sqrt{\frac{\sum_{i=\Delta t-T}^{\Delta t+T}{\left(S\left(i\right)-\overset{ˉ}{S}\right)}^{2}}{n}}$$

(8) $$\mathrm{Maximize}:\;\Delta t_{end}-\Delta t_{start},\sigma_{t}\geq \sigma_{\min},\;t\in \left[\Delta t_{end}-\Delta t_{start}\right]$$

(9) $$\Delta t_{end}-\Delta t_{start}\geq 0.7\;s$$

In Equation (7), *n* represents the number of samples within Δt and $\overset{ˉ}{S}$ is the average value of samples. Meanwhile, in order to avoid other interference, we set the duration of each sliding window to 0.7 s. $\left[\Delta t_{end}-\Delta t_{start}\right]$ is the detected region.

*Data augmentation*. Since the neural network needs a large amount of data, we use the data augmentation scheme to increase the diversity of data and the robustness of the system. Specifically, we refer to the data augmentation method in [37] and scale the original data along the timestamp by different multiples $\beta \in \left\{0.6,0.8,1.2,1.4\right\}$. In addition, Gaussian noise is added to the original data and the scaled data to make the data have some errors, so as to better simulate the real situation.

#### 2.3.3. Deep Learning Module

After preprocessing, the data is passed to the deep learning module, which is processed by the neural network to realize the feature extraction function, and the person detection function is realized through the classifier of the output layer. This section mainly introduces the VGG convolutional neural network [38].

**Figure 9:**
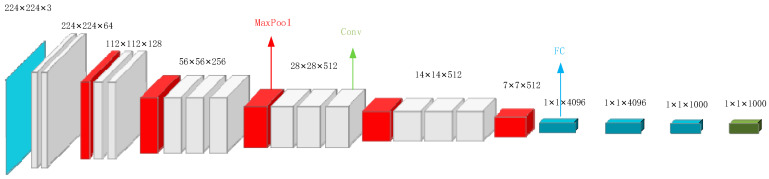
VGG Network structure.

*VGG Convolutional Neural Network*. As deep learning has become an important development direction in machine learning, the use of convolutional neural network (CNN) to solve image classification and recognition problems has gradually become mainstream. As a feedforward neural network, CNN combines an artificial neural network with deep learning, and trains the weights in the network through the improved back propagation algorithm based on gradient to realize the deep learning algorithm. However, VGG, as one of the classical convolutional neural networks, has three convolutional layers, three pooling layers and a fully connected layer. By stacking multiple small-size 3 × 3 convolution filters to replace the large convolution kernel, the calculation can be effectively reduced, and the regularization effect and classification accuracy can be improved. The network structure is shown in Figure 9.

1. Convolutional layer. The convolutional layer is mainly used for gait image feature extraction and recognition. VGG uses a uniform 3 × 3 convolutional filter to overlay the input matrix, and then carries out the inner product with the overlaid input submatrix, plus a certain bias value to obtain an element of the output matrix. After sliding with a certain step size, the above steps are repeated to obtain the entire output matrix. The specific calculation method is as follows:
  (10) $$c_{n}^{i}=\underset{i\in M}{\sum }c_{mn}^{i-1}w_{mn}^{i}+b_{n}^{i}\;$$
  where $c_{n}^{i}$ represents the $n^{th}$ element of the output matrix, $c_{\mathrm{mn}}^{i-1}$ is the element of the $m^{th}$ row and $n^{th}$ column of the input matrix, $w_{mn}^{i}$ is the weight of the $m^{th}$ row and $n^{th}$ column, $b_{n}^{i}$ is the bias value, and M represents the set of input matrices.
2. Pooling layer. The essence of pooling is sampling, which occurs after convolution, and its process is similar to convolution. The pooling kernel size used by VGG is 2 × 2, and the step size is two. The pooling layer selects a certain way to reduce the dimension and compress the input features to eliminate redundant data information, speed up the operation and prevent the algorithm from overfitting. Pooling is similar to the sliding operation of the convolution operation.

In the operation of convolution and pooling, the size of the input image and the output image should meet the following relationship:

(11) $$o=\frac{i+2b-f}{s}+1$$

Here, i represents the size of the input image, b is the size of the input image boundary fill, f is the filter size, s is the sliding step size, and o is the size of the output image.

3. Fully connected layers. This layer is the same as the traditional neural network, that is, there is a connection relationship between any two meta-neural units of the input layer and the output layer.

*Classifier*. The SoftMax classifier is used in the output layer to classify the input data. The SoftMax function has the form:

(12) $$S\left(i\right)=\frac{e^{-\theta_{i}x}}{\sum_{j=1}^{n}e^{-\theta_{j}x}}\;$$

θ is the parameter to be evaluated, n is the total number of categories, and x is the input data. Softmax makes $S\left(i\right)$ between [0, 1]. When performing classification, Softmax follows the following principles:

(13) $$\left(y^{\left(i\right)}=n|x^{\left(i\right)};W\right)=\left[\begin{array}{l} S(y^{\left(i\right)}=1|x^{\left(i\right)};W)\\ S(y^{\left(i\right)}=2|x^{\left(i\right)};W)\\ \vdots \\ S(y^{\left(i\right)}=n|x^{\left(i\right)};W) \end{array}\right]=\frac{1}{\sum_{j=1}^{n}e^{w_{j}x^{\left(i\right)}}}\cdot \left[\begin{array}{l} e^{w_{1}x^{\left(i\right)}}\\ e^{w_{2}x^{\left(i\right)}}\\ \vdots \\ e^{w_{n}x^{\left(i\right)}} \end{array}\right]$$

$S\left(y^{\left(i\right)}=n|x^{\left(i\right)};W\right)$ is the hypothesis function, $x^{\left(i\right)}$ is the input, $y^{\left(i\right)}$ is the class label, W is the weight, where $w_{n}x^{\left(i\right)}$ is the input of the Softmax layer. Finally, the formula (13) returns the probability of each class for each input.

## 3. Results

In this section, we first introduce the experimental setup of the system along with the required experimental parameters, and then show the experimental evaluation results under different conditions.

### 3.1. Experimental Setup

**Figure 10:**
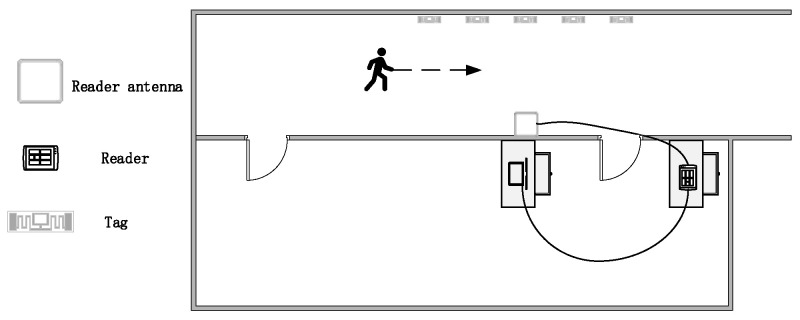
Experimental scene.

Experimental environment: Figure 10 shows our experimental scene, where the tag group is attached to the wall on one side of the corridor, and the reader is positioned on the wall on the other side and perpendicular to the middle tag. The direct tag spacing is 0.7 m, and the vertical distance between the reader and the tag is 2 m. When conducting person detection, the user starts to walk through the corridor in the reader’s receiving range. When the reader receives the signal of the tag, it transmits the tag to the PC through Ethernet for data reception and identification.

**Figure 11:**
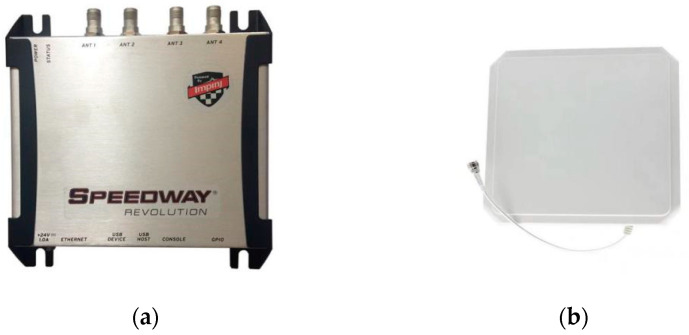
Experimental equipment. (**a**) Impinj Speedway R420 reader; (**b**) UHF circularly polarized antenna.

Hardware environment: As shown in Figure 11, the hardware facility contains an Impinj Speedway R420 reader and an RFID UHF circularly polarized antenna (9 dBi gain; the receiving range is 120°). The operating frequency of the reader is 920.875 MHz, the tag uses a 73 × 20 mm UHF flexible anti-metal UR107 tag and a laptop computer is used for data reception.

Software Facilities: We run the model on a Lenovo computer equipped with 2.5 GHz AMDR7 and 16 gigabytes of memory for data acquisition and preprocessing, the RFID reader is connected to the laptop via an Ethernet cable, and the low-level reader protocol (LLRP) is used for communication. The method is implemented in C, and the neural model we designed is implemented in Python.

Dataset: We collected a total of 7500 data samples from 10 volunteers (all 10 volunteers were adults, four women and six men, and two of the four women were the same height, two were different in height, and six men were different in height). To evaluate the effectiveness of the person detection, volunteers were asked to walk in a normal walking manner, and each group of volunteers crossed the reader reception area for about 3–6 s, and then the next group of volunteers continued sampling.

### 3.2. Detection of Different Step Lengths

**Figure 12:**
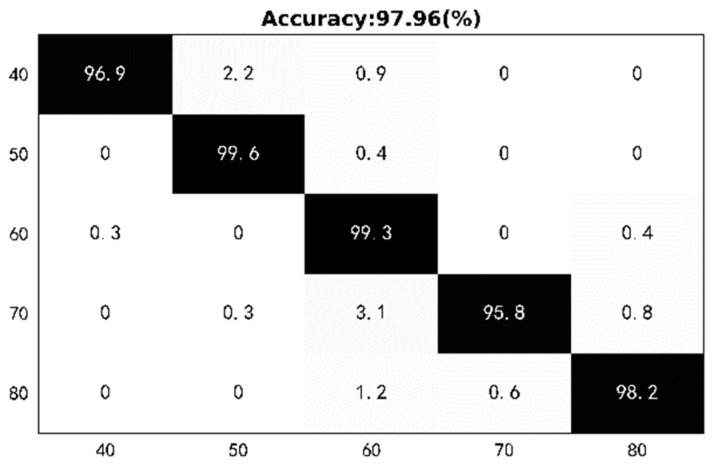
Confusion matrix of out-of-sync distances test.

In order to evaluate the accuracy of different step lengths detection, we divide the step lengths into five groups (40 cm, 50 cm, 60 cm, 70 cm, 80 cm), and all users walk in a normal way. A total of 150 samples were used as test data for a new user, and the remaining samples were used as training data. Figure 12 illustrates the detection performance with the five different step lengths. The experimental results show that the detection accuracy of RF-Detection for new users reaches 97.96%. In addition, based on the step length, we can calculate the approximate height of the user at this step length.

### 3.3. Effect of Different Speeds

**Figure 13:**
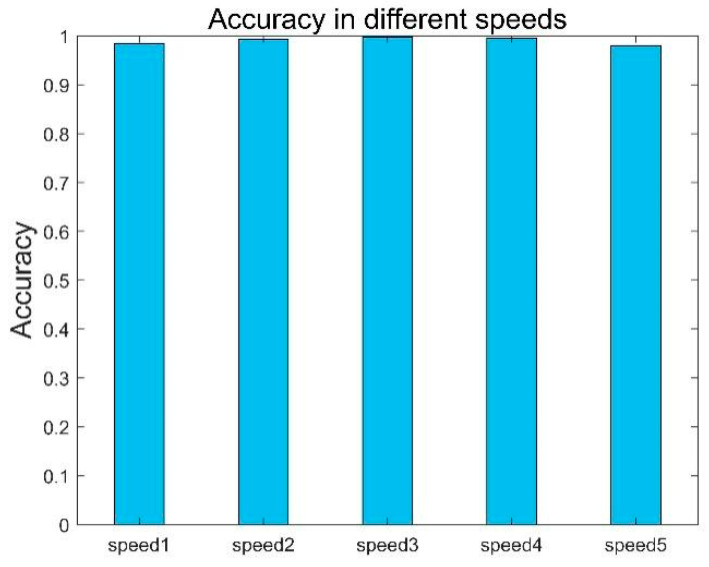
Performance at different speeds.

In some cases, the user’s pace may be affected by his mood and other factors. In order to explore the influence of speed on step length, we set up four groups of comparative experiments for analysis. First, we took 30 samples under experimental conditions as standard test data, and then we invited 12 volunteers, who were different from those in the experiment, to be divided into four groups according to different walking speeds for comparative experiments, with 30 test data from each group. The experimental results are shown in Figure 13, and we find that the accuracy for speeds 2, 3 and 4 is relatively average, reaching more than 99%. However, the accuracy for speed 5 is low, only 98%, which may be due to the unstable signal caused by the user walking too fast.

### 3.4. Effect of Different Disturbances

**Figure 14:**
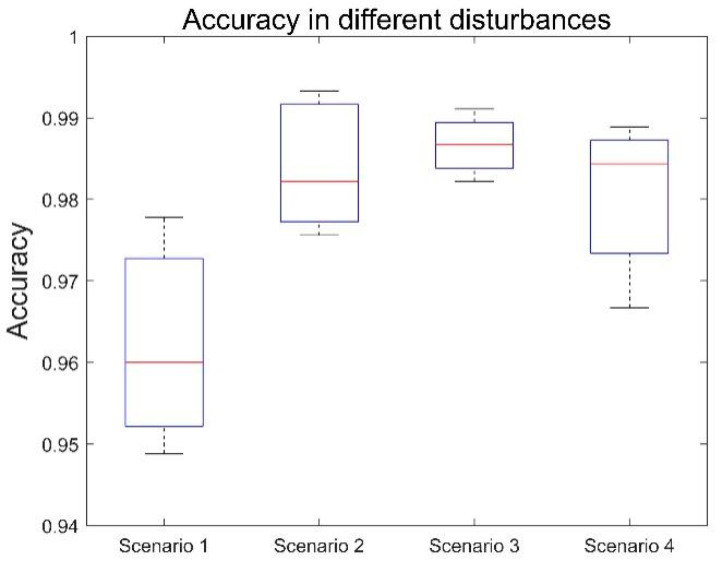
Performance under different interferences.

Electronic products such as RFID tags are susceptible to interference from metals and other wireless signals, even if they have been designed and manufactured with this in mind. Metals will cause eddy currents around superfrequency RFID tags and readers, thus reducing the overall efficiency of RFID electromagnetic fields. In addition, surrounding objects will also reflect RFID signals and cause interference. In order to verify the performance of RF-Detection under interference factors, we set up four interference scenes to simulate the state of the crowd waiting at a zebra crossing for comparison (excluding the cycling crowd). In Scene 1, the user carries a handbag. In Scene 2, the user carries a phone and puts it in his pocket. In Scene 3, the user holds a phone in his hand. In Scene 4, the user plays a mobile phone while completing the experiment. The same amount of experimental data as in Experiment C was obtained for each scene. The experimental results are shown in Figure 14. For experimental scene 1, the accuracy of the experiment is 96%. For experimental scene 2, the accuracy of the experiment is 98.22%. For experimental scene 3, the accuracy of the experiment is 98.89%. For experimental scene 4, the accuracy of the experiment is 98.44%. The reason for the low accuracy of Scene 1 is that the handbag is located near the knee in the experiment, which is not conducive to the collection of gait data. The overall experiment shows that RF-Detection has a strong anti-environmental-interference capability.

### 3.5. Continuous Person Detection

**Figure 15:**
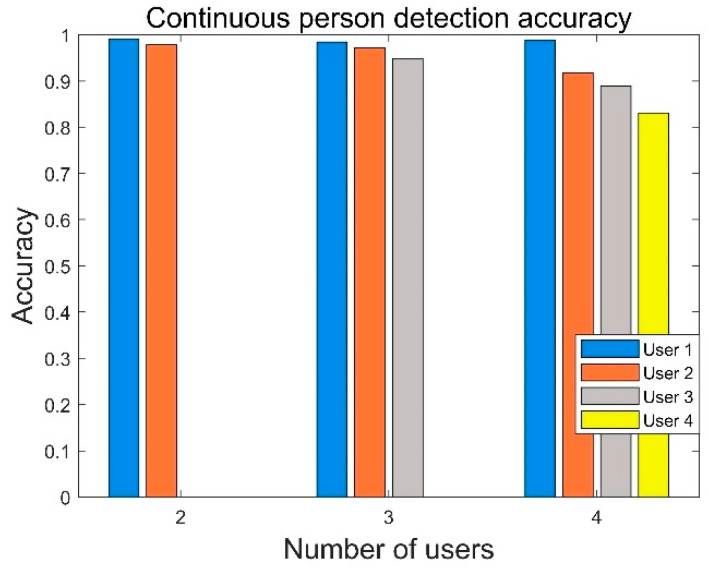
Performance of continuous person detection.

RF-Detection can perform the detection classification of continuous persons. For continuous persons, three groups of experiments were conducted, where two, three and four people passed through the detection area continuously, and the step lengths in each group were not all the same. The experimental results are shown in Figure 15. It can be seen from the experiment that the accuracy for the first person of the segmentation is often the highest, and with an increase in the number of people, the accuracy rate significantly decreases. When the number of experimenters was three, the accuracy remained in a relatively stable state, and the average accuracy was 96.81%. When there were four experimenters, the accuracy of the experiment was significantly reduced, even though the last member performed the same steps as the first member, and the accuracy was only 83.11%. This shows that with the increase in persons, the existing experimental equipment cannot distinguish between people with different step lengths well. In addition, this problem may also be caused by the segmentation method and the short experiment time.

## 4. Discussion

Given the limitations of existing conditions, the application of RF-Detection in real life is still a great challenge. Firstly, existing experimental data cannot be well applied to specific program requirements, and more experimental data need to be collected. At the same time, the existing segmentation methods show poor results in continuous person detection, which makes it difficult to play a good role for scenes with large human flow, such as sidewalk and bus congestion detection. In subsequent experiments, we will focus on improving the segmentation strategy, and plan to summarize a new method by exploring the segmentation method used in [39,40]. Secondly, although RF-Detection shows good performance in experiments, its accuracy may be greatly reduced when applied in different environments because a model trained in one environment is difficult to apply in another environment. We plan to collect experimental data from multiple environments and develop a set of environment-independent detection models to be applied to the system through adversarial training. Finally, we will continue to improve the deep learning method. In this plan, we will draw on the Deep Convolution Generative Adversarial Networks (DCGAN) adversarial neural network, which cannot only expand the data, but also realize the distinction between the user domain and the environment domain, which can get better results.

In future work, in addition to optimizing RF-Detection itself, we will also apply it to real-world scenes for testing. For example, to simulate an overloading detection scene of persons on a bus, the system is placed at the door of the laboratory to simulate the entrance of the bus, and the experimenter passes the entrance to realize the boarding operation. After that, the system calculates the number of people on the bus by segmentation to complete the scene experiment. By conducting an age-range testing experiment, we know that there is a direct and indispensable relationship between adult age and height. In the following work, we will use experiments to verify this conjecture, and calculate the age range of the experimenters through the step length, so as to achieve age distinction. Simulating human rescue experiment scenes, and testing the number of persons indoors in different experimental environments (office, corridor, underground garage, etc.), will also be carried out to complete the experiments. We believe that RF-Detection can achieve relatively perfect results in the direction of person detection.

## 5. Conclusions

In this paper, we propose RF-Detection, an RFID-based person detection system that can realize the classification of a user’s height based on their step length information, and at the same time can realize the detection work for continuous users. RF-Detection automatically completes user detection from the spatio-temporal information obtained from RFID tags. Moreover, through a series of preprocessing works such as signal smoothing and signal segmentation, the original signal can be extracted as a feature in the VGG convolutional network to achieve accurate person identification and detection. Extensive experiments prove that RF-Detection can achieve an average accuracy of 98.93%, and the average accuracy for new users is 97.96%. Given its good performance as well as a wide range of application scenes, we believe that RF-Detection can be a good booster to promote the development of passive sensing.

## Data Availability

Not applicable.
